# Enhancing cancer care with improved checkpoint inhibitors: a focus on PD-1/PD-L1

**DOI:** 10.17179/excli2024-7783

**Published:** 2024-10-29

**Authors:** Vickram A S, Saghya Infant Shofia, A. Saravanan, Vidhya Lakshmi Sivakumar, Packiyam Thamarai, Manikandan Sivasubramanian, Shivani Chopra, Hitesh Chopra

**Affiliations:** 1 Department of Biotechnology, Saveetha School of Engineering, Saveetha Institute of Medical and Technical Sciences, Chennai, India; 2 Department of Biosciences, Saveetha School of Engineering, Saveetha Institute of Medical and Technical Sciences, Chennai - 602105, Tamil Nadu, India; 3 Center for Research Impact & Outcome, Chitkara College of Pharmacy, Chitkara University, Rajpura, 140401, Punjab, India

**Keywords:** checkpoint inhibitors, PD-1, PD-L1, cancer, immunotherapy

## Abstract

The emergence of checkpoint inhibitors targeting the PD-1/PD-L1 axis marks a paradigm shift in cancer therapy, offering a novel avenue for enhancing patient outcomes. This review examines the structural and functional dynamics of PD-1 and PD-L1 while exploring the clinical implications of current PD-1/PD-L1 monoclonal antibodies. Highlighting recent advancements, this paper delves into the promising results from combination therapies that present a multifaceted attack on tumor progression. Despite the success observed across various cancer types, challenges such as immune resistance remain. Future considerations are discussed with an emphasis on the need for further clinical studies, aiming to refine and broaden the curative potential of PD-1/PD-L1 inhibitors in oncology. This review postulates that ongoing research and innovative approaches could significantly enhance cancer care, making immunotherapy an even more central strategy in the fight against cancer.

See also the graphical abstract(Fig. 1).

## The Paradigm Shift in Cancer Therapy with Checkpoint Inhibitors

For decades, traditional methods of cancer treatment like surgery, chemotherapy, and radiation have held the upper hand in the world of cancer therapy. While each has its own merits and drawbacks, their primary aim has been to directly eliminate cancer cells, often with repercussions for healthy tissue and serious side effects for patients. In recent times, a significant shift has occurred, marking a new era in the fight against cancer: the emergence of immune checkpoint inhibitors. These therapies harness the power of our body's immune system to combat cancer, bringing a fresh perspective to the table (Maawy and Ito, 2019[60]; He and Xu, 2020[33]). 

The development of these groundbreaking medications has completely transformed the world of cancer treatment, revolutionizing the way we combat the disease. With these innovative drugs, we can now harness the power of the immune system to fight cancer more efficiently and with greater consideration for the patient's well-being (Ribas and Wolchok, 2018[84]). A crucial component in this breakthrough is the understanding of immune checkpoints - molecules within the immune system that can either intensify a signal (known as co-stimulators) or weaken a signal (known as co-inhibitors). Unfortunately, cancer cells often exploit these checkpoints, evading detection and attack from the body's own immune cells (Dine et al., 2017[21]; Darvin et al., 2018[20]). 

An exciting finding in the field of cancer immunotherapy has been the identification of immune checkpoints, particularly CTLA-4 and PD-1/PD-L1, which serve as key regulators in protecting tumors from immune attacks (Qin et al., 2019[79]). This groundbreaking discovery has paved the way for the development of monoclonal antibodies known as checkpoint inhibitors, designed to target these proteins and disable their "off" signals. By doing so, these inhibitors empower the immune system to recognize and eliminate cancer cells (Shiravand et al., 2022[90]).

The recent development and usage of drugs aiming at PD-1 and PD-L1 have brought about monumental breakthroughs in the management of various cancers, such as melanoma, non-small cell lung cancer, kidney cancer, bladder cancer, and head and neck cancers (Lee et al., 2022[48]). These successes are abundant, as patients have seen remarkable and sustained remissions, along with a significant boost in their chances of survival. However, this progress has not come without hurdles and obstacles to overcome (Wang et al., 2021[105]). 

The immune landscape and tumor microenvironment are complex and not all patients are affected in the same way by checkpoint inhibitors. As a result, there has been a surge of research aimed at unravelling the reasons behind resistant tumors and discovering biomarkers that can accurately forecast the individuals most likely to benefit from these therapies (Larkin et al., 2019[47]). Despite these challenges, the remarkable clinical efficacy of checkpoint inhibitors has sparked a renaissance in the field of oncology. These treatments not only enhance survival rates for specific cancers, but also provide a better quality of life for numerous patients by minimizing side effects when compared to traditional therapies (Barrueto et al., 2020[6]).

Exploring the intricacies of molecular and cellular processes, scientists have uncovered the inner workings of checkpoint pathways and their vital role in immune regulation (Shao et al., 2023[89]). Among the various types of immune cells, T cells possess the remarkable ability to selectively seek out and eliminate cancerous cells. However, in the presence of a tumor, these cells can become suppressed and "exhausted" by inhibitory signals (Sun et al., 2023[96]). To counteract this, inhibitors targeting key checkpoints such as PD-1/PD-L1 and CTLA-4 have emerged as crucial tools in the field of immuno-oncology, effectively reactivating these exhausted T cells (Immuno-oncology checkpoint inhibitors, 2022[37]). 

The continuous improvement of these medications has been greatly aided by advancements in genetic sequencing and bioinformatics. This enables a more personalized and tailored approach to treatment. By delving into the genetic composition of a patient's tumor, there is potential to anticipate their response to checkpoint inhibitor therapy. This leads to the creation of individualized and efficient care plans, as demonstrated by the research (Sun et al., 2023[97]). Moreover, the incorporation of checkpoint inhibitors with other forms of treatment, such as targeted therapies, chemotherapy, and radiation, has resulted in a powerful synergy. This has shown to be more effective in addressing cancer than utilizing any single therapy alone (Li et al., 2023[52]). 

As we observe the ongoing evolution of checkpoint inhibitors, scientists are delving into innovative avenues to develop the next generation of these medicines, striving to bolster their effectiveness, overcome barriers to treatment, and minimize negative consequences (Li et al., 2019[51]). Ongoing clinical studies are actively investigating combination therapy approaches, alternative dosing methods, and novel targets that go beyond the traditional PD-1/PD-L1 and CTLA-4 pathways (Yi et al., 2022[120]).

The use of checkpoint inhibitors marks a groundbreaking change in cancer treatment, but we are only at the start of this revolution. This breakthrough opens the door to a promising new era in medicine, where harnessing the power of the immune system can potentially overcome cancer. As science progresses and more individuals reap the benefits of these innovative therapies, the ultimate aim is to make checkpoint inhibitors a commonplace and essential aspect of cancer care. The goal is to provide patients with longer, healthier lives, and transform what was once feared as an insurmountable disease into something manageable or even curable.

Chang et al. (2021[12]) reviewed FDA approvals of PD-1/PD-L1 inhibitors over six years, examining the reasons for approval, regulatory pathways, and use of companion diagnostics. They emphasized the efficacy of these drugs while noting the moderate response rates and the need for more research (Table 1(Tab. 1); References in Table 1: Forde et al., 2018[25]; Li et al., 2019[53]; Lin et al., 2018[55]; Mansh, 2011[61]; Mazieres et al., 2020[63]; Migden et al., 2018[65]; Motzer et al., 2018[66]; Paz-Ares et al., 2020[77]; Reck et al., 2020[82]; Rocco et al., 2022[85]; Rolfo et al., 2017[86]; Shiravand et al., 2022[90]; Shirley, 2018[91]; Wei et al., 2018[107]; Wu et al., 2021[112]). Chen et al. (2021[15]) conducted a meta-analysis of 91 clinical trials to assess the effectiveness of PD-1/PD-L1 inhibitors in various clinical scenarios, quantifying overall response rate, time to response, and duration of response across different cancers, treatment lines, drug combinations, and regimens. Zhang et al. (2024[123]) performed a meta-analysis of 69 randomized controlled trials to investigate cardiovascular toxicity risks associated with PD-1/PD-L1 inhibitors in solid tumors, finding elevated risks of hypertension, hypotension, arrhythmia, and myocarditis, especially when combined with chemotherapy or CTLA-4 inhibitors. Finally, this current review offers a comprehensive overview of PD-1/PD-L1 inhibitors, encompassing their mechanism of action, clinical implications, combination therapies, challenges such as immune resistance, and future research directions, with a focus on the potential of combination therapies and a qualitative analysis of their mechanisms and clinical impact.

## The PD-1/PD-L1 Axis: Mechanisms of Action and Pathway Modulation

The PD-1/PD-L1 axis is an essential pathway in the immune system that acts as a key checkpoint, controlling the immune response and upholding immune balance. This axis encompasses the interaction between the programmed cell death protein-1 receptor found on T-cells and its ligand, PD-L1, present on both cancer cells and cells within the tumor microenvironment (Patsoukis et al., 2020[76]). 

Discovered in 1992, PD-1 (also known as CD279) is a protein involved in regulating the immune system. Similar to proteins like CD28, CTLA4, and ICOS, PD-1 plays a role in immune function. Its structure includes both an extracellular segment and cytoplasmic tail that are responsible for carrying out signaling functions (Parvez et al., 2023[74]). PD-1 is found on a range of activated immune cells, particularly those infiltrating tumors. Transcription factors such as NFAT, NOTCH, FOX O1, and IRF9 control the expression of the PD-1 gene. While PD-1 is crucial for maintaining immune balance and tolerance, it can also hinder the body's natural response to cancer cells, potentially aiding the progression of tumors (Chen et al., 2023[14]).

The essential protein, PD-L1 serves a crucial function in regulating immune responses. It is produced by a variety of cells, including macrophages, T cells, B cells, dendritic cells, and certain epithelial cells, especially in the presence of inflammation. This protein assists tumors in evading immune surveillance, as highlighted by (Han et al., 2020[32]). Interestingly, the upregulation of PD-L1 is stimulated by interferon-gamma, which can worsen conditions like ovarian cancer. However, when the IFN-γ receptor is blocked, PD-L1 expression decreases, affecting pathways in acute myeloid leukemia. Additionally, chronic infections can lead to T cell exhaustion, characterized by increased PD-1 expression. This is closely linked to a demethylated promoter region and the influence of transcription factors, such as FOXO1, as outlined by Wu et al. (2019[114]). Moreover, the secretion of IFN-γ by both NK cells and T cells not only amplifies the expression of PD-L1 on tumor cells, but also triggers the JAK1, JAK2, and STAT1 pathways, ultimately bolstering the tumor's ability to evade immune surveillance (Padmanabhan et al., 2022[73]). 

On top of this, the binding of PD-L1 to its target receptors spurs on cancer cell growth and survival pathways, potentially instigating epithelial-mesenchymal transition and conferring stem cell-like properties that fuel tumor advancement, as observed in cases of renal cancer (Zhou et al., 2023[129]). The immune system is a complex network, and within it, two important proteins, PD-1 and PD-L1, play a critical role in keeping it in balance. These proteins are part of the PD-1/PD-L1 pathway, which helps regulate the body's immune response and maintain a state of immune tolerance. This pathway acts like a checkpoint, safeguarding against autoimmune reactions and ensuring a proper regulation of immune reactions (Yi et al., 2021[119]).

Examining the PD-1/PD-L1 pathway more closely reveals its significance. First and foremost, PD-1 is found on T cells, a crucial component of our immune system responsible for fighting off infections and cancer. On the other hand, PD-L1 is often present on cells within the tumor environment and some normal tissues (Ghosh et al., 2021[29]). When PD-1 on the surface of T cells binds with PD-L1 on other cells, such as cancer cells or antigen-presenting cells, this triggers an inhibitory signal. Essentially, this signal tells the T cells to reduce their activity, which is a normal process in immune regulation. This helps prevent T cells from attacking our own cells and maintains a balanced immune system (Jiang et al., 2019[39]). Cancer cells have a sneaky way of avoiding detection by the immune system. They do so by overexpressing a protein called PD-L1, which binds to another protein on T cells called PD-1, effectively shutting down the immune response (Dong et al., 2018[22]).

Thankfully, there is a way to disrupt this process and unleash the immune system's power to fight back. This can be done through the use of immune checkpoint inhibitors, a type of drug that blocks the PD-1 and PD-L1 interaction. By inhibiting PD-L1, these medications restore the T cells' ability to attack the cancer cells, providing a potential therapeutic intervention (Wu et al., 2022[113]). The role of the PD-1/PD-L1 pathway in regulating immune tolerance is crucial, as it requires a delicate balance between targeting harmful cells and preserving healthy ones. This intricacy has sparked significant research and has led to the development of advanced cancer treatments that aim to enhance the immune system's ability to fight cancer (Zhou et al., 2022[128]). Modulating the PD-1/PD-L1 pathway involves targeted efforts to disrupt the communication between PD-1 receptors on T-cells and PD-L1 ligands, which are frequently exaggerated on cancer cells and within the tumor microenvironment. This approach aims to bolster the immune system's capacity to combat cancer (Wu et al., 2019[114]).

Immune checkpoint inhibitors are a class of drugs designed to specifically target and suppress proteins involved in regulating the immune system, such as PD-1 or PD-L1. Through this mechanism, they disrupt the inhibitory signals that typically suppress the immune response, particularly in T cells fighting against cancerous tumors (Lao et al., 2022[46]). Monoclonal antibodies (mAbs) play a crucial role in drug therapy, as they specifically target either PD-1 or PD-L1 to prevent their interaction. For instance, anti-PD-1 mAbs bind to PD-1 receptors on T-cells, while anti-PD-L1 mAbs target PD-L1 ligands present on cancer cells or other cells in the tumor microenvironment (Li et al., 2021[54]). This mechanism makes mAbs an effective treatment option for inhibiting the growth and spread of cancer. 

### Enhanced T-cell response

**Figure 2:**
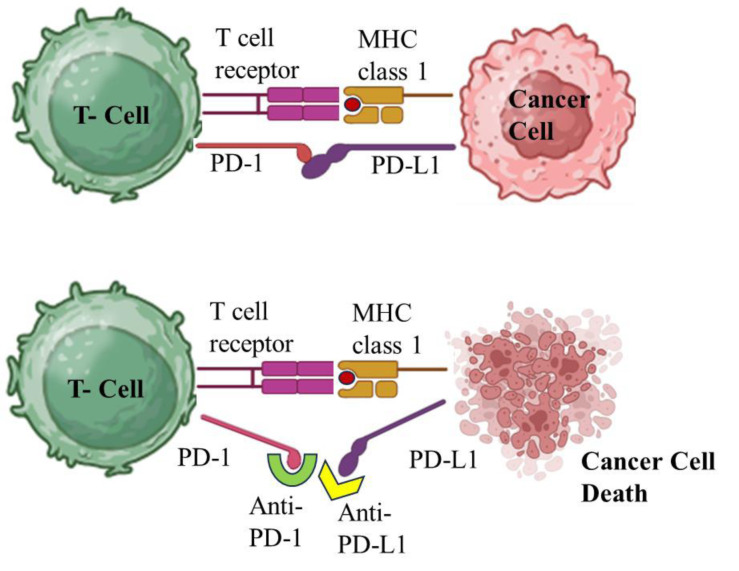
Reactivation of T-cell function by PD-1/PD-L1 inhibitors and the role of T-cell receptors, antigens, and MHC

By inhibiting the PD-1/PD-L1 interaction, T-cells are able to break free from the restrictive effects of this pathway. This crucial step can reverse the state of T-cell exhaustion and revitalize their ability to multiply and carry out their function. As a result, the immune system can launch a stronger and more effective attack against cancer cells (Barrueto et al., 2020[6]). When it comes to optimizing treatment outcomes, utilizing a combination of therapeutic approaches has become increasingly common in the use of PD-1/PD-L1 inhibitors. In order to enhance their efficacy, these inhibitors are often paired with other treatments such as chemotherapy, radiation, or other forms of immunotherapy. By combining these treatments, a more conducive environment can be created for the immune system to effectively battle cancer cells (Lee et al., 2022[48]). The advent of PD-1/PD-L1 pathway modulators has revolutionized the approach to treating numerous cancers, bringing about substantial changes in clinical outcomes. Ongoing clinical trials and studies are constantly uncovering the potential of these modulators in various cancer types, with the ultimate goal of enhancing treatment strategies and identifying the most suitable candidates for optimal outcomes (Liu et al., 2022[56]). Figure 2(Fig. 2) depicts the mechanism of PD-1/PD-L1 Inhibitors for reactivation of T cell function.

The manipulation of the PD-1/PD-L1 pathway signifies a calculated change in the way we approach cancer therapy, harnessing the natural power of the immune system to combat disease. This has emerged as a critical focus in the field of oncology research (Liu et al., 2022[58]). Ultimately, the PD-1/PD-L1 axis plays a crucial role in the immune checkpoint system by maintaining a delicate balance between immunity and self-tolerance, preventing harmful immune reactions while also regulating the body's ability to combat cancer. In light of our understanding of this critical axis, we have developed immunotherapies that specifically target the interaction between PD-1 receptors and PD-L1 ligands, effectively restoring the immune system's ability to identify and destroy cancer cells. These groundbreaking treatments, including monoclonal antibodies and checkpoint inhibitors, have completely revolutionized the field of cancer treatment by significantly boosting the body's immune response against various types of cancer, representing a major milestone in the field of oncology (Somu et al., 2024[93]; Supriya et al., 2024[99]).

## Refinements in PD-1/PD-L1 Blockade: Recent Advances and Developments

PD-1 and PD-L1 inhibition is a form of immunotherapy that has transformed the treatment of various cancers. This blockade works by targeting a key regulatory pathway of the immune system, which is often exploited by cancer cells to evade the immune response (Festino et al., 2018[23]). The refinements and recent developments in PD-1/PD-L1 blockade includes firstly the clinical efficacy in melanoma. Advanced melanoma treatment has seen a significant shift with the advent of anti-PD-1 and anti-PD-L1 therapies. The clinical efficacy of PD-1/PD-L1 blockade in the treatment of melanoma represents one of the most significant advancements in the management of this difficult-to-treat cancer (Zhao et al., 2020[124]). 

These treatments have led to improved survival rates and are now a cornerstone in the management of metastatic melanoma (Table 2(Tab. 2); References in Table 2: Anand et al., 2023[2]; Bulgarelli et al., 2021[9]; Conry et al., 2018[18]; Garutti et al., 2022[27]; Heinzerling et al., 2019[34]; Kakadia et al., 2018[40]; Okiyama and Tanaka, 2022[71]; Shyam Sunder et al., 2023[92]; Willsmore et al., 2021[108]; Wojtukiewicz et al., 2021[109]; Xiong et al., 2022[116]). Prior to the introduction of these immunotherapies, patients with metastatic melanoma faced a very poor prognosis, with median sur vival times of less than one year (Sundararajan et al., 2022[98]). The emergence of checkpoint inhibitors like nivolumab (anti-PD-1) and ipilimumab (anti-CTLA-4) has radically improved survival (Gellrich et al., 2020[28]). Data shows that dual therapy with these agents can lead to a 58 % survival rate at three years, which is a significant improvement from historical outcomes (Wolchok et al., 2017[110]). Clinical trials have demonstrated the efficacy of these therapies in increasing patient survival rates and managing disease progression. Key clinical trials that established the efficacy of PD-1 blockers in melanoma include the CheckMate-067 trial (Bari et al., 2022[5]).

This study compared nivolumab alone, ipilimumab alone, and the combination of both drugs in patients with advanced melanoma. Results showed that nivolumab alone, as well as in combination with ipilimumab, significantly improved overall survival rates compared to ipilimumab alone (Rager et al., 2022[80]). 

### Monotherapy vs. combination therapy

While monotherapies using PD-1 inhibitors like pembrolizumab or nivolumab have shown effectiveness, combination therapy regimens pairing nivolumab with ipilimumab have demonstrated even greater benefits in overall response rates and survival, albeit with increased toxicity (Carreau and Pavlick, 2019[10]). Importantly, a subset of melanoma patients treated with PD-1/PD-L1 inhibitors seems to achieve long-term remission, effectively controlling the disease over extended periods. These prolonged responses suggest that PD-1 blockade could potentially offer a form of cure in some cases of advanced melanoma where few treatment options existed before (Chen et al., 2021[15]).

### Checkpoint inhibitor mechanism

These drugs work by targeting immune checkpoints. These checkpoints are molecules on certain immune cells which, when activated, can diminish the immune response (Wu et al., 2019[115]). In the case of PD-1 (programmed death-1), a receptor on T cells, it interacts with PD-L1 (ligand 1), often overexpressed on tumor cells. The essence of PD-1/PD-L1 inhibitors' action is to prevent the exhaustion of T cells, a state where T cells gradually lose their ability to combat cancer cells effectively. When PD-1, present on the T cells, interacts with PD-L1 on tumor cells, the immune response is downregulated, allowing cancer cells to grow and spread. PD-1/PD-L1 inhibitors disrupt this interaction, thereby reinvigorating the T cells to attack and kill tumor cells (Zila et al., 2021[131]; Ai et al., 2020[1]). Initially used for melanoma, PD-1/PD-L1 blockade is now being extended to treat other cancer types. Through ongoing clinical trials, the use of these inhibitors is approved for various malignancies, including Hodgkin lymphoma, bladder cancer, and many more. Emerging evidence suggests that they might also be effective against traditionally hard-to-treat cancers (Twomey and Zhang, 2021[102]).

### Comparison with previous treatments

The emergence of PD-1/PD-L1 blockade therapy represented a paradigm shift in the treatment of metastatic melanoma, surpassing the effectiveness of older treatments like chemotherapy, which had limited impact on long-term survival and often resulted in significant side effects without improving overall survival (Shafi, 2023[88]). Recent research is focusing on the potential synergistic effects of PD-1/PD-L1 inhibitors when combined with other cancer therapies, such as chemotherapy, radiation, and other immunotherapies. These combinations may enhance the immune system's response and address the issue of resistance to single-agent immunotherapy (Yi et al., 2022[120]). Advanced stages of melanoma, which have traditionally been challenging to treat and associated with very poor outcomes, are now being managed more effectively due to these immunotherapeutic agents. The durable responses observed in some patients suggest that these treatments can lead to lasting control of the disease, which was previously unattainable with conventional therapies (Fujimura et al., 2020[26]).

Biomarkers are biological molecules that provide information about a patient's cancer and how it may respond to certain therapies. The search for reliable biomarkers that can predict which patients will benefit from PD-1/PD-L1 blockade is of great interest (Ou et al., 2022[72]). Tumor PD-L1 expression is one of the most extensively studied biomarkers for these inhibitors. The logic is straightforward: if a tumor expresses PD-L1 and thereby weakens the immune response, blocking that pathway could release the "brakes" on the immune system, allowing T cells to target the tumor more effectively. However, PD-L1 expression levels can vary widely and may be influenced by the tumor microenvironment, and not all patients with PD-L1 positive tumors respond to treatment (Michielin et al., 2020[64]; Zappasodi et al., 2018[122]).

Tumor PD-L1 expression and other factors such as mutational burden and immune cell infiltration within the tumor environment have been evaluated as potential biomarkers (Ma et al., 2022[59]). 

### Tumor mutational burden

High tumor mutational burgen (TMB) has been associated with better responses to immunotherapy, including PD-1/PD-L1 inhibitors. The theory is that tumors with a higher number of mutations may present more neoantigens, which can be recognized as foreign by the immune system, thus targeting the cancer cells more effectively (Chen et al., 2019[13]).

### Immune cell infiltrate

The presence and type of immune cells within and around the tumor, often called the immune contexture, can also serve as a biomarker. A "hot" tumor, characterized by a heavy infiltration of T cells, may be more likely to respond to PD-1/PD-L1 blockade than a "cold" tumor with fewer immune cells present (Huang et al., 2021[36]).

However, the predictive value of these markers is not absolute, and researchers are working on identifying and validating additional biomarkers to better select patients for PD-1/PD-L1 inhibitor treatment (Rager et al., 2022[80]). Ongoing research aims to refine and validate biomarkers for PD-1/PD-L1 inhibitor response. This includes looking at combinations of biomarkers and exploring other potential indicators such as specific gene expression patterns, microsatellite instability, and other immune checkpoint molecules (Cohen et al., 2016[17]). 

### Combining biomarkers

Researchers are investigating the potential of combining different biomarkers to improve the predictive accuracy for treatment response (Song et al., 2023[94]). By using a panel of biomarkers, it might be possible to get a more comprehensive understanding of the tumor and its relationship with the immune system, leading to better patient stratification and personalized treatment strategies (Islam et al., 2024[38]).

### Dynamic changes

It is also recognized that biomarkers can change over time, and what is present at initial diagnosis may not reflect the tumor biology after treatment begins. There is interest in assessing biomarker status at multiple points during treatment to allow dynamic adjustment of therapeutic strategies (Ribas, 2012[83]). 

### Integrating biomarkers with clinical variables

Another promising area is the integration of biomarker profiles with traditional clinical variables, such as stage of disease and prior treatment history, to develop more robust predictive models for patient response to PD-1/PD-L1 therapies (Auslander et al., 2018[4]).

### Overcoming resistance

Some tumors do not respond to PD-1/PD-L1 blockade, or they develop resistance after an initial response. Understanding the mechanisms of resistance is crucial for refining these therapies. Studies are exploring the tumor microenvironment, genetic mutations, and alternative immune checkpoints to find ways to overcome this challenge (Nowicki et al., 2018[70]). 

### Mechanisms of resistance

Cancer cells may employ various tactics to resist immune checkpoint inhibitors. These mechanisms include alterations in the tumor microenvironment that create an immunosuppressive state, mutations in genes involved in the PD-1/PD-L1 pathway, expression of alternative immune checkpoints, or changes in metabolic pathways affecting immune cell function (Lei et al., 2020[50]). 

### Research in overcoming resistance

To improve the efficacy of PD-1/PD-L1 inhibitors, research is focusing on understanding these resistance mechanisms. Studies are exploring the combination of checkpoint inhibitors with other forms of cancer therapy, such as vaccines, targeted therapies, or adoptive cell transfer, to enhance the immune response to cancer cells (Andrews et al., 2020[3]; Goh et al., 2023[30]).

Clinicians are also experimenting with different dosing schedules, combining PD-1/PD-L1 inhibitors with other types of immune checkpoint blockers, and using predictive biomarkers to tailor treatment approaches for each patient as ways to combat resistance (Watson et al., 2020[106]). The goal is to extend the benefits of immunotherapy to more patients and enhance the durability of responses, transforming how we treat various cancers and improving patient outcomes (Zhu et al., 2021[130]). In summary, PD-1/PD-L1 blockade has significantly advanced the field of oncology, offering hope to many patients with hard-to-treat cancers. Ongoing research is focused on not only expanding the use of these therapies to more tumor types but also refining the approach to enhance efficacy, minimize side effects, and ultimately, improve patient outcomes (Mukherjee et al., 2022[67]).

## Evaluating the Efficacy: Clinical Trial Insights and Patient Outcomes

Evaluating the efficacy of PD-1/PD-L1 inhibitors involves assessing their therapeutic impact on cancer, particularly in their role as immunotherapies for conditions like melanoma. The PD-1/PD-L1 pathway is a critical immune checkpoint that, when exploited by cancer cells, can lead to immune evasion. Inhibitors that block this pathway can enhance the immune system's ability to fight cancer (Tang et al., 2018[100]). The evaluation process for the efficacy of PD-1/PD-L1 inhibitors typically includes the following phases of clinical trial (Tang et al., 2018[101]):

- Phase I Trials: To determine the safety, tolerable dose range, and side effects of PD-1/PD-L1 inhibitors when administered to humans. Initial observations regarding efficacy may also be made.
- Phase II Trials: To assess the effectiveness of the drugs in patients with specific types of cancer and further evaluate the safety profile.
- Phase III Trials: To confirm the effectiveness of the inhibitors, monitor adverse side effects, and compare their performance to standard treatments.

In PD-1/PD-L1 inhibitor trials, several outcome measures are employed to evaluate the efficacy of the treatment. These typically include the objective response rate, which is the proportion of patients whose tumor size is reduced by a predefined amount for a specified period (Schmidt et al., 2020[87]). Progression-free survival is also measured, indicating the duration patients live without the cancer worsening. Overall survival is a critical metric as well, representing the time from the start of treatment that patients are still alive. In addition to these traditional clinical endpoints, quality of life assessments is often included in trials to gauge the treatment's impact on the daily functioning and well-being of patients. These various outcome measures collectively inform the effectiveness and therapeutic benefit of PD-1/PD-L1 inhibitors in clinical settings (Qin et al., 2024[78]).

**Figure 3:**
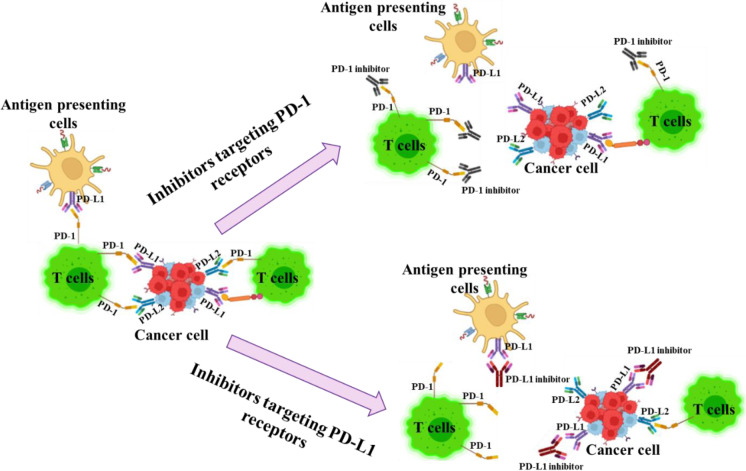
Mechanism of action: PD-1/PD-L1 inhibitors and their role in enhancing anti-tumor immunity

Despite often showing promise in clinical trials, there are limitations associated with PD-1/PD-L1 therapy that must be taken into account when evaluating efficacy. Response rates vary with the type of cancer and stage of disease, and some patients may not respond at all or may develop resistance over time (Ou et al., 2022[72]). Furthermore, the balance between immune activation and the risk of immune-related side effects needs to be carefully managed. Biomarkers, such as PD-L1 expression levels, might help predict response but are not always definitive indicators of treatment success. These challenges and considerations highlight the complexity of effectively using PD-1/PD-L1 inhibitors as part of cancer treatment (Zhao et al., 2022[125]). Figure 3(Fig. 3) depicts the mechanism of action of PD-1/PD-L1 inhibitors and their role in enhancing anti-tumor immunity.

The expression levels of PD-L1 on tumor cells or in the tumor microenvironment have been studied as potential predictive biomarkers for response to PD-1/PD-L1 inhibitors. However, the predictive power of PD-L1 expression is not absolute, and some patients with high PD-L1 may not respond to therapy while others with low or no expression may still benefit (Ribas, 2012[83]). Other biomarkers, such as tumor mutational burden and mismatch repair deficiency, are also under investigation for their potential to predict responsiveness to immunotherapy. As the field of oncology progresses, genetic profiling of tumors continues to advance, potentially leading to more precise methods for predicting which patients will benefit most from PD-1/PD-L1 inhibitors (Betof Warner et al., 2020[7]).

PD-1/PD-L1 inhibitors can offer sustained advantages to those undergoing treatment, as certain patients may achieve lasting responses that extend the period of cancer management. This underlines the importance of long-term follow-up studies, which are essential for understanding the full impact of these treatments over time (Yi et al., 2018[118]). Assessing the duration of response helps to evaluate how long the benefits of the therapy last, and whether they translate to improved overall survival and quality of life. The longevity of a favorable response to PD-1/PD-L1 therapy is a key factor in considering the long-term efficacy of these immunotherapeutic agents in the management of various cancers (Herbst et al., 2020[35]). 

Real-world effectiveness of PD-1/PD-L1 inhibitors is assessed through observational studies conducted post-approval, which collect data from a broader and more diverse patient population than those typically enrolled in clinical trials (Kumaresan et al., 2022[45]). These studies provide valuable insights into the actual impact of the treatments in routine clinical practice, including their influence on overall survival and long-term health-related quality of life. Such real-world data can reveal effectiveness in a setting that mirrors everyday healthcare conditions, helping to understand how these immunotherapeutic agents perform outside the tightly controlled constraints of clinical research (Cramer-van Der Welle et al., 2021[19]; Pasello et al., 2020[75]).

While evaluating efficacy, it's equally important to monitor the safety profile and manage potential adverse events that can range from mild skin reactions to severe immune-related complications (Su et al., 2020[95]). Adverse events associated with PD-1/PD-L1 inhibitors can impact a range of organs and systems, reflecting the widespread role of the immune system in the body. The management of these side effects is critical, as they can be life-threatening and may require treatment discontinuation. Pharmacovigilance and post-market surveillance are essential to continually assess the safety profile of these drugs and provide updated guidelines for their use (Kessler and Pandruvada, 2023[41]). 

This vigilance helps to balance the potential benefits of therapy against the risks, ensuring patient safety while striving to achieve the best possible treatment outcomes. The high cost of PD-1/PD-L1 inhibitors can impact their accessibility and overall cost-effectiveness. Health economics studies are essential to consider the value these treatments bring relative to their price (Martins et al., 2019[62]). Evaluating the efficacy of PD-1/PD-L1 inhibitors is a complex, multi-step process that takes into account clinical trial data, long-term health outcomes, patient quality of life, and broader health economic factors. Advancements in this field continue to refine our understanding of patient selection and treatment optimization (Yin et al., 2023[121]).

Incorporation of Patient-Reported Outcomes (PRO) is becoming increasingly important in the evaluation of PD-1/PD-L1 inhibitors. These outcomes can give insight into the patients' perspective on symptoms, side effects, and overall impact on their quality of life (King-Kallimanis et al., 2019[43]). Capturing this information is critical as it reflects the real-world experience of patients and helps healthcare providers to tailor treatments to individual needs (Nishijima et al., 2019[69]). Moreover, PRO data contribute to treatment evaluations beyond standard clinical measures, ensuring that patient satisfaction and subjective well-being are factored into the overall assessment of therapy success. The routine inclusion of PROs in clinical trials and post-marketing surveillance is essential for a holistic understanding of treatment efficacy and patient-centered care (Tykodi et al., 2018[103]). 

**Figure 4:**
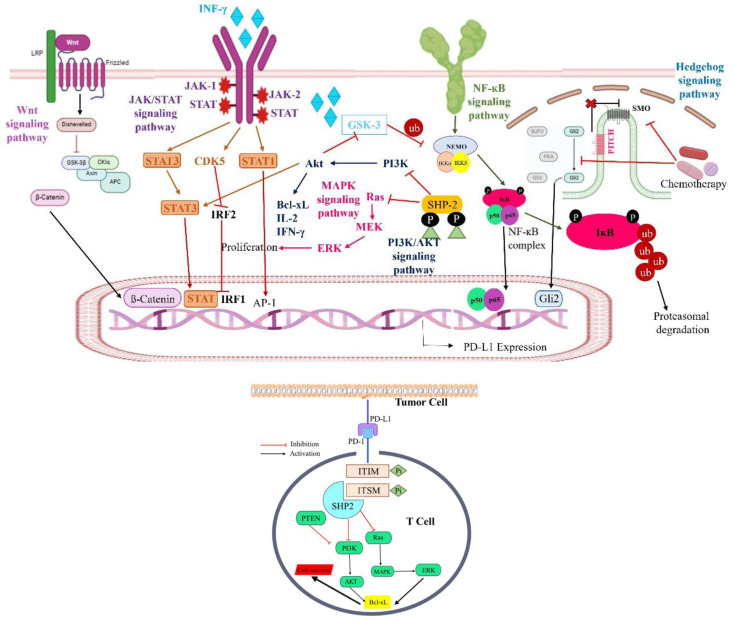
(a) The regulation of PD-1/PD-L1 expression involves multiple pathways. The PI3K/AKT pathway, MAPK pathway, JAK/STAT pathway, WNT pathway, NF-κB pathway, and Hedgehog (Hh) pathway collectively stimulate the expression of the PD-1/PD-L1 axis. (b) Impact of PD-1/PD-L1 immune checkpoint engagement on T-cell fate. The interaction between PD-1 and PD-L1 triggers phosphorylation within PD-1's ITIMs and ITSMs, prompting the association with SHP-2 phosphatase. This interaction inhibits the PI3K/AKT and Ras/MAPK/ERK pathways, culminating in T-cell apoptosis.

The future of PD-1/PD-L1 inhibitor efficacy evaluation may lie in a more personalized approach to treatment, where therapy is tailored based on individual patient characteristics and genetic profiles. Evaluating the efficacy of PD-1/PD-L1 inhibitors requires a comprehensive approach that combines clinical trial data, real-world outcomes, and biomarker research for patient selection. Long-term follow-up is crucial for assessing sustained treatment success. The aim is to personalize cancer immunotherapy by integrating clinical, molecular, and genetic data, optimizing treatment efficacy and reducing side effects. PRO are also vital to ensure treatments meet patient needs and improve quality of life (Fitzsimmons et al., 2023[24]). Figure 4(Fig. 4) illustrates the multiple pathways for the regulation of PD-1/PD-L1 expression.

## Critical Discussion of the Limitations and Potential Side Effects Associated with PD-1/PD-L1 Checkpoint Inhibitors

PD-1/PD-L1 checkpoint inhibitors have revolutionized cancer treatment, but they are not without limitations and potential side effects. While PD-1/PD-L1 checkpoint inhibitors have demonstrated remarkable success in some cancer types, a significant challenge remains in the substantial proportion of patients who do not derive clinical benefit from these therapies. This inherent or acquired resistance to PD-1/PD-L1 blockade can arise from a complex interplay of factors, hindering the efficacy of these treatments and necessitating further investigation. Tumor cells with insufficient PD-L1 expression may not be effectively targeted by PD-1/PD-L1 inhibitors (Nowicki et al., 2018[70]). While PD-L1 expression is often used as a biomarker for patient selection, its predictive value remains imperfect, and alternative biomarkers are needed. The presence of tumor-infiltrating lymphocytes is crucial for the antitumor activity of checkpoint inhibitors. Tumors with low TIL (tumor-infiltrating lymphocytes) counts, often termed "cold tumors," may not respond effectively to PD-1/PD-L1 blockade. Strategies to enhance T-cell infiltration into the tumor microenvironment are being explored. Tumors can employ various immune-suppressive mechanisms beyond the PD-1/PD-L1 axis. These mechanisms, which may involve other immune checkpoints, immunosuppressive cytokines, or regulatory T cells, can limit the efficacy of PD-1/PD-L1 inhibitors (Zhou et al., 2021[127]). Tumors are often composed of diverse cell populations with varying levels of PD-L1 expression and sensitivity to immune checkpoint blockade. This heterogeneity can contribute to treatment resistance and disease relapse. Even among initial responders, some patients may develop acquired resistance over time. This can occur through the emergence of new immune-suppressive mechanisms or the selection of resistant tumor cell clones. A particularly concerning limitation of PD-1/PD-L1 blockade is the potential for hyperprogression. This phenomenon, characterized by accelerated tumor growth following treatment initiation, is observed in a subset of patients and can lead to rapid clinical deterioration. The underlying mechanisms of hyperprogression remain poorly understood, and its prediction is challenging. Further research is needed to identify biomarkers that can predict hyperprogression and to develop strategies to mitigate its risk (Champiat et al., 2018[11]; Han et al., 2020[31]).

Immune-related adverse events (irAEs) are a significant concern with PD-1/PD-L1 inhibitors. These irAEs arise from the enhanced immune activity induced by checkpoint blockade, which, while targeting cancer cells, can also disrupt the delicate balance of the immune system, leading to unintended attacks on healthy tissues and organs (Zheng and Wei 2021[126]). Consequently, irAEs can affect a wide range of organ systems, and their severity can vary significantly. Common immune-related adverse events (irAEs) associated with PD-1/PD-L1 inhibitors can affect various organ systems. Dermatologic irAEs include rash, pruritus, and vitiligo. Gastrointestinal irAEs can manifest as colitis, diarrhea, or hepatitis. Endocrine irAEs may include thyroid dysfunction, hypophysitis, and type 1 diabetes. Pulmonary irAEs such as pneumonitis can also occur. Neurologic irAEs can present as neuropathy or myasthenia gravis (Chuzi et al., 2017[16]; Kumar et al., 2017[44]; Wang et al., 2019[104]; Lee et al., 2020[49]; Ramos-Casals et al., 2020[81]; Yang et al., 2020[117]; Naidoo et al., 2023[68]). While most irAEs are mild to moderate and manageable with appropriate interventions, some can be severe and life-threatening. The management of irAEs often involves a careful balance between controlling the immune-related inflammation and preserving the antitumor efficacy of the checkpoint inhibitors (Brahmer et al., 2018[8]). Corticosteroids, such as prednisone, are commonly used to suppress the immune response and manage irAEs. However, prolonged use of corticosteroids can have significant side effects and may compromise the antitumor activity of the checkpoint inhibitors. In cases of severe or refractory irAEs, other immunosuppressive agents, such as infliximab or mycophenolate mofetil, may be necessary. Supportive care measures, such as pain management, fluid replacement, and nutritional support, are essential for managing the symptoms of irAEs and promoting patient comfort (Liu et al., 2019[57]; Khan and Monzon 2020[42]). Careful monitoring for the development of irAEs is crucial, and prompt management is essential to minimize their impact on patient outcomes. Early recognition and appropriate intervention can often prevent irAEs from becoming severe or life-threatening (Wood, 2019[111]).

## Conclusion: The Future of PD-1/PD-L1 Inhibition in Oncology Practice

Immunotherapy, particularly the use of PD-1/PD-L1 inhibitors, has become a pivotal aspect of oncological treatment, revolutionizing the management of various cancers. The future of these inhibitors in clinical practice is poised for significant developments. The range of cancers treatable with PD-1/PD-L1 inhibitors is set to broaden as research progresses, potentially standardizing these therapies for a more extensive array of malignancies. A critical factor in the widespread adoption of these agents is the identification of predictive biomarkers to discern responsive patient populations, as the current benefit to patients is variable. Comprehending and overcoming resistance to PD-1/PD-L1 therapies is imperative, and ongoing studies focus on the underlying mechanisms that some tumors exploit to elude these treatments. Optimal management of immune-related adverse events associated with these inhibitors is also a priority, as the prevalence of toxicities may increase with more patients being treated. There are unanswered questions regarding the ideal length of treatment and the feasibility of continuing therapy post-progression, which researchers are actively exploring. 

The integration of PD-1/PD-L1 inhibitors with other therapeutic options-including chemotherapy, targeted agents, and additional immunotherapies-is an exciting prospect that could lead to enhanced outcomes. The field is progressively moving towards personalized medicine, aiming to tailor immunotherapeutic interventions to individual tumor profiles and immune system characteristics, which is expected to boost the effectiveness of these agents significantly. Lastly, the financial aspect of PD-1/PD-L1 inhibitor therapies is a growing concern; ensuring these are cost-effective and accessible forms part of the larger dialogue in healthcare economics. In essence, PD-1/PD-L1 inhibition is set to expand its role in oncology, with scientific advances, practical strategies, and novel clinical trials contributing to its evolution, thereby advancing the paradigm of cancer care and fortifying the hope for improved patient prognoses.

## Figures and Tables

**Table 1:**
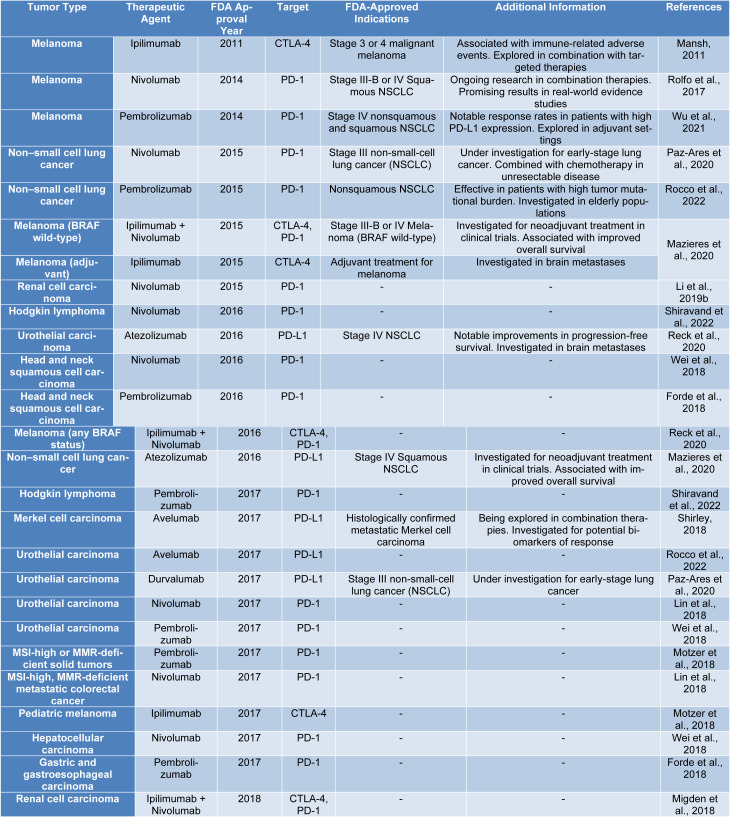
FDA-approved immune checkpoint inhibitors and their indications across various tumor types

**Table 2:**
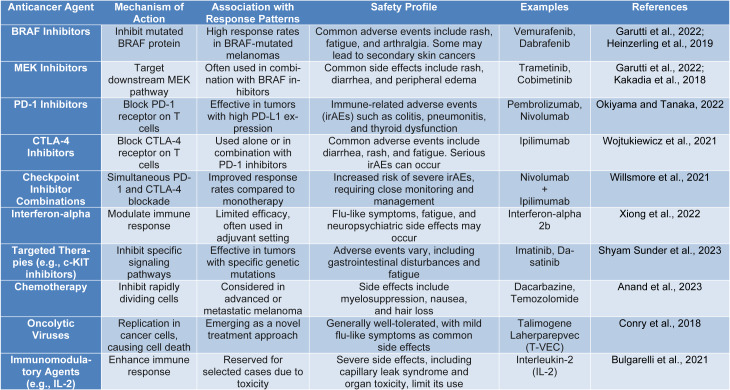
Mode of action of anticancer agents in melanoma and their correlation with response patterns and safety characteristics

**Figure 1:**
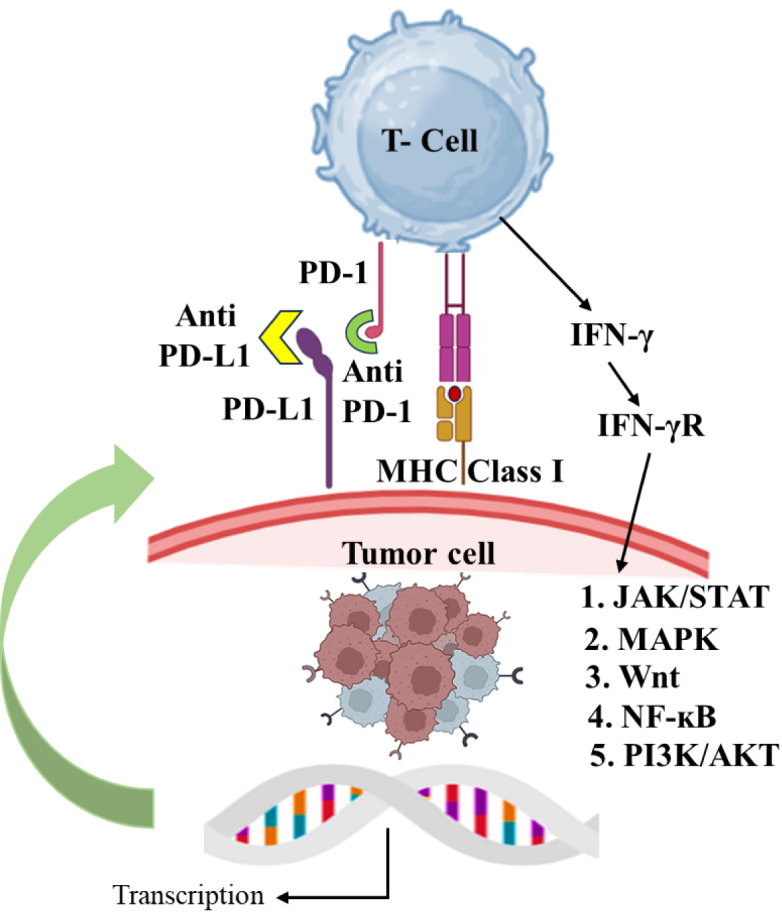
Graphical abstract
